# Pericarditis Epistenocardica or Dressler Syndrome? An Autopsy Case

**DOI:** 10.1155/2015/215340

**Published:** 2015-07-09

**Authors:** Alessandro Feola, Noè De Stefano, Bruno Della Pietra

**Affiliations:** ^1^Department of Experimental Medicine, Second University of Naples, Via Luciano Armanni 5, 80138 Naples, Italy; ^2^Unit of Histology and Anatomical Pathology, A.O.R.N. “San Giuseppe Moscati”, Contrada Amoretta, 83100 Avellino, Italy

## Abstract

Postinfarction pericarditis can be classified as “early,” referred to as pericarditis epistenocardica, or “delayed,” referred to as Dressler syndrome. The incidence of postinfarction pericarditis has decreased to <5% since the introduction of reperfusion therapies and limitation of infarct size. We report on a 57-year-old man who suffered sudden cardiac death as a result of acute myocardial infarction. Autopsy revealed an area of previous infarction and fibrinous pericarditis related to the previous infarction, leading to a diagnosis of Dressler syndrome.

## 1. Introduction

Acute pericarditis is defined as inflammation of the pericardium with production of fibrinous inflammatory exudate. It is diagnosed in approximately 0.1% of hospitalized patients and in 5% of patients admitted to the emergency department with noncardiac chest pain [1]. Acute pericarditis has several potential causes. Idiopathic pericarditis is the most common cause; others include infection (viral, e.g., Coxsackie A/B; bacterial, e.g., Staphylococcus; mycobacterial, fungal, and parasitic), systemic autoimmune inflammatory diseases (e.g., connectivitis, vasculitis, rheumatoid fever, and granulomatosis), pathologies of the surrounding organs, cardiac damage (e.g., Dressler syndrome, postpericardiotomy syndrome, postheart transplant, and pulmonary embolism), and pericardial trauma. Less frequent causes include neoplastic pericarditis, radiation or drugs, congenital renal pathologies, and endocrine metabolic pathologies (e.g., myxedema and goiter) [2]. At the clinical level, it is possible to distinguish among acute (onset <6 weeks), subacute (6 weeks–6 months), and chronic forms (>6 months) [3]. We present a patient with pericarditis resulting in sudden cardiac death.

## 2. Case Presentation

**Figure 1:**
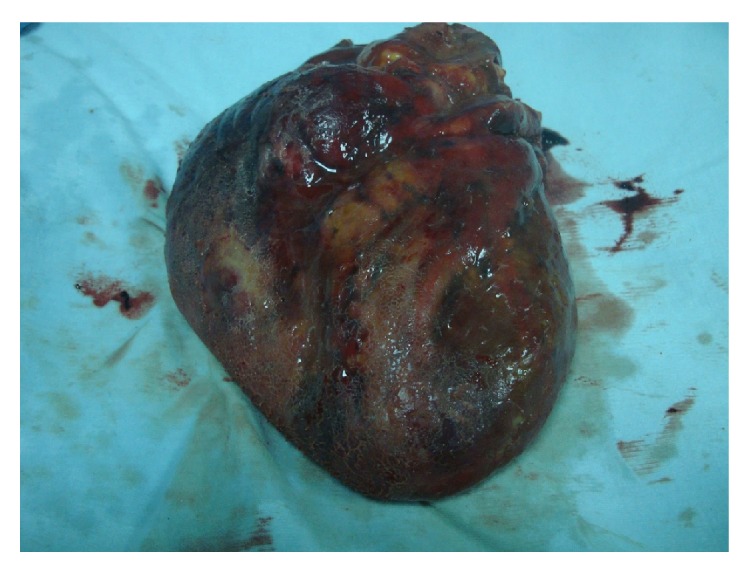
Macroscopic view of the heart prior to formalin fixation.

**Figure 2:**
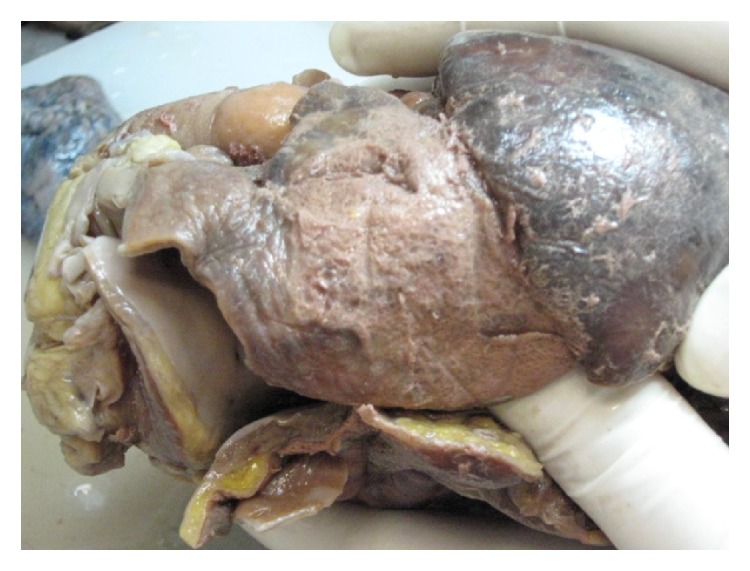
Macroscopic view of areas with fibrinous pericarditis (following formalin fixation).

**Figure 3:**
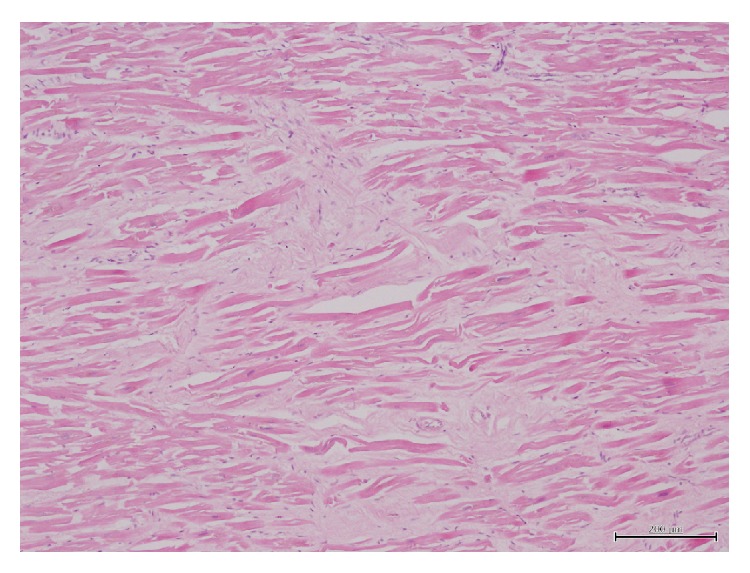
Hematoxylin/eosin-stained section of myocardium illustrating areas of myocardial sclerosis (×100).

**Figure 4:**
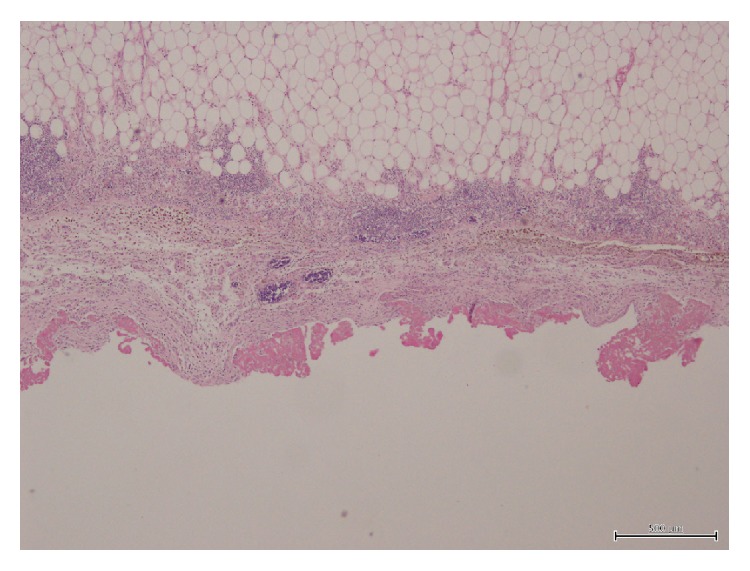
Hematoxylin/eosin-stained view of fibrinous pericarditis (×40).

**Figure 5:**
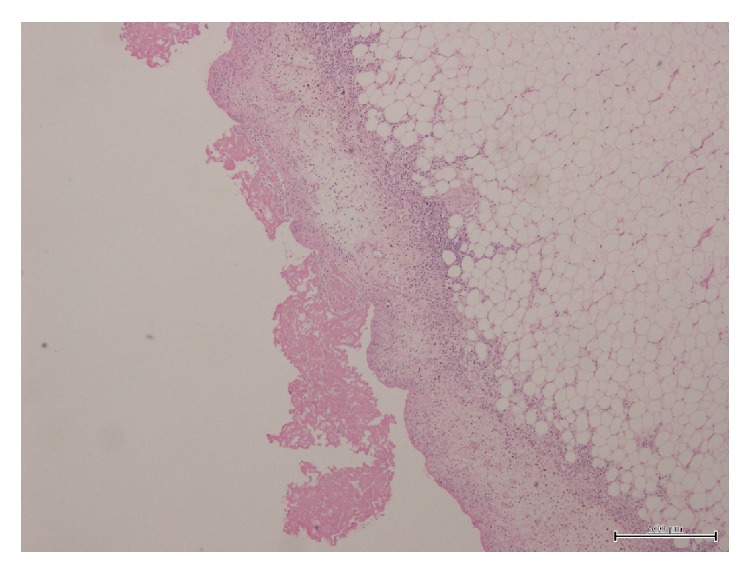
Hematoxylin/eosin-stained view of fibrinous pericarditis (×40).

A 57-year-old man was found dead in the hallway of his house. His family members reported seeing him 24 hours earlier, when he appeared to be in good health, except for a slight pain in his right ankle. However, they also reported that he had complained of intense chest pain approximately 1 month before. His medical history consisted of untreated hypertension. An autopsy performed 72 hours after discovery was noted. All the organs appeared congested and macroscopic examination showed evidence of interlobular pleurisy. The heart after extraction and fixation in 10% buffered formalin weighed 724 g, measured 15 × 13 × 5 cm, and had a truncated conical shape with a brick-red color. The epicardium was characterized by a fine granular layer on its surface with a “bread and butter” appearance, particularly at the level of the atrial regions and the cardiac apex (Figures 1 and 2). The cardiac cavities were slightly dilated and lined by a smooth and shiny endocardium. The thicknesses of the left ventricle, right ventricle, and interventricular septum were 23 mm, 6 mm, and 22 mm, respectively. The atrioventricular valve apparatus appeared normal. The mitral valve was two fingers wide and the tricuspid valve was three fingers wide, both with elastic valve flaps and no signs of nodular formations or calcium deposits. The aortic valve flaps were slightly thickened and focally affected by a few yellowish plaques. In cross section, the myocardial tissue showed a soft-elastic consistency and was dark red in color, with darker variegations appearing compacted. The coronary sinus was free from obstruction. Fat striations were observed in the aortic sinus (sinus of Valsalva). The coronary vessels had focal atherosclerotic plaques resulting in reduced lumen diameter, particularly in the anterior descending branch of the left coronary artery. Cardiac tissue was prepared and embedded in paraffin wax, and 4 *μ*m thick cross sections were cut and stained with hematoxylin/eosin, by standard methods. Plurifocal signs of myocardial sclerosis were detected in the wall of the left ventricle and the interventricular septum (Figure 3). Few lymphocytic perimyofibrillar microinfiltrations were observed, as well as several nonspecific postmortem alterations, including fragmentatio cordis, and structural disarrangement of the myocytes. Localized lipofuscin pigment deposits were occasionally observed in the myocytes, mainly in the perinuclear area, and moderate adipose replacement with no fibrous aspects involving the right ventricular myocardium. No alterations were observed in the endocardium. The serous cavity demonstrated histological features that could be attributed to fibrinous pericarditis (Figures 4 and 5). Samples collected from the proximal coronary branches revealed coronarosclerosis, subendothelial intimal sclerosis, and sclerotic calcified plaques. There was no evidence of significant alterations of the subepicardial distal vessels in the sections examined. Biological samples collected at autopsy were subjected to systematic toxicological analysis using headspace gas chromatography (flame-ionization detection), gas chromatography-mass spectrometry, and liquid chromatography-tandem mass spectrometry but were negative for alcohol and for most common illicit drugs and pharmaceuticals.

## 3. Discussion

The pericardium can respond to acute myocardial infarction (MI) in different ways, particularly by pericardial effusion or pericarditis [4]. Postinfarction pericarditis can be classified as either “early,” referred to as pericarditis epistenocardica, or “delayed,” referred to as Dressler syndrome [5]. Before the advent of thrombolysis, the reported incidence of clinical infarction-associated pericardial involvement was 7–23%, with much higher rates detected during autopsy [6]. However, the incidence of postinfarction pericarditis decreased to <5% since the introduction of reperfusion therapies and the limitation of infarct size [6].

Pericarditis epistenocardica, which initially manifests with pain and a pericardial rub, usually occurs within the first three days after a transmural infarction [5, 7]. Transmural infarctions result from transmural necrosis with inflammation affecting the adjacent visceral and parietal pericardium [8]. In such cases, the classic electrocardiography (ECG) changes of pericarditis are usually not apparent, suggesting a relapse of the subepicardial lesion and/or an increase in ischemia [9]. Although chest X-ray and echocardiography are not diagnostic in the event of a localized pericardial reaction, they become useful, especially the latter, in the event of a pericardial effusion. The diagnosis is based on clinical suspicion, fever, pleuritic chest pain, and the presence of an effusion by echocardiography [10].

Dressler syndrome occurs after MI. Prior to the reperfusion era, its reported incidence was 1–5% of patients with acute MI [11]. However, its advent of thrombolysis and widespread use of heparin have reduced the incidence of this syndrome [12]. When present, it arises 2 weeks after an MI presumably due to an autoreactive immune mechanism similar to postcardiac injury syndrome [10, 13]. Typical symptoms include pleuritic chest pain with low-grade fever. A pericardial rub is present in 50% of patients [14]. Chest X-ray may show a pleural effusion and/or enlargement of the cardiac silhouette. An ECG often demonstrates ST-elevation and T-wave changes typical of acute pericarditis [10].

The two syndromes differ dramatically in several areas. Dressler syndrome is more paucisymptomatic with few manifestations, such as fever, malaise, and chest pain [15, 16]. Diagnostic ECG changes of pericarditis epistenocardica require a transmural MI in order to injure the visceral pericardium but Dressler syndrome does not [15].

In the current case, death was caused by a hyperacute infarct. As there was no histological evidence of granulocytes at the myocardial level and only bands with myosclerosis, there was no evidence of pericarditis caused by the most recent infarction (Figure 3). However, the granulocytes were present at the level of the pericardium, evidence of pericarditis due to the previous infarction (Figures 4 and 5).

In conclusion, Dressler syndrome appeared to be the most likely diagnosis, given the presence of fibrinous pericarditis with evidence of a previous MI and interlobular pleurisy. A pulmonary genesis of the pleurisy was excluded based on negative macroscopic and microscopic examination of the lungs.
